# Real-life efficacy of immunotherapy for Sézary syndrome: a multicenter observational cohort study

**DOI:** 10.1016/j.eclinm.2024.102896

**Published:** 2024-10-29

**Authors:** Belinda A. Campbell, H. Miles Prince, Julia J. Scarisbrick

**Affiliations:** a Department of Radiation Oncology, Peter MacCallum Cancer Centre, Melbourne, Australia; b Sir Peter MacCallum Department of Oncology, University of Melbourne, Parkville, Australia; c Department of Clinical Pathology, University of Melbourne, Parkville, Australia; d Department of Haematology, Peter MacCallum Cancer Centre and Royal Melbourne Hospital, Melbourne, Australia; e Department of Dermatology, University Hospital Birmingham, Birmingham, United Kingdom

We congratulate the authors on their recent report on outcomes of patients treated for Sezary syndrome (SS) over a 20-year period.

The low utilisation of allogeneic stem-cell transplantation (AlloSCT) (only 17 (5.0%) patients) differs from our recent international study (13.5%)^1^ but confirms earlier observations of significant treatment heterogeneity between jurisdictions.^2^ We are interested to understand the per-centre recruitment, and if all centres offered AlloSCT? Particularly as prognostic benefits from ‘high-volume centres’ are reported from the National Cancer Database,^3^ and AlloSCT is the only therapy offering curative potential for SS.^4^ We also ask what influence AlloSCT had on survival in this cohort, and if mogamulizumab improved survival independently from AlloSCT?

One challenge to assessing time-to-next-treatment (TTNT) is the risk of over-stating the last treatment line, hence, the “TTNT-clock” must stop when end-of-life care commences.^5^ We ask if this was used in these analyses? The median TTNT of mogamulizamab (24 months) exceeds previous reports (13 months).^1^^,^^6^ It would be helpful to know how many patients received mogamulizumab as last-line, also, the TTNT of mogamulizamab excluding re-exposures, thus avoiding re-treatment bias in patients experiencing treatment-efficacy and tolerance.

We would be interested to understand the impact of combination therapies. Do these data support the significantly longer TTNT from combinations over monotherapies, as previously reported?^1^ Further, to inform optimal treatment sequencing, how did treatment-line impact TTNT? Both combination and treatment-line were significantly impactful for photopheresis in our data,^1^ such analyses may help determine the efficacy of therapeutic combinations and improve outcomes in SS.

## Contributors

All authors contributed to the writing of this letter.

## Declaration of interests

BA Campbell: Advisory board & Honoraria received from Kyowa Kirin.

HM Prince: Advisory boards &/or Honoraria received from Kyowa Kirin, MundiPharma, Mallinkrodt, Innate Pharma.

JJ Scarisbrick: Advisory boards &/or Honoraria received Recordati, Helsinn, Kyowa Kirin, Mallinkrodt, Takeda, 4SC; President of the International Society for Cutaneous Lymphomas (ISCL).
